# Caregiving Intensity and Caregiver Burden Among Dementia Caregivers: The Moderating Roles of Social Support

**DOI:** 10.1093/geroni/igaa057.234

**Published:** 2020-12-16

**Authors:** Ling Xu, Yiwei Liu, Hui He, Noelle Fields, Chen Kan, Dorothea Ivey

**Affiliations:** 1 The University of Texas at Arlington, Arlington, Texas, United States; 2 Central University of Finance and Economics, Beijing, China; 3 Xiangtan University, Xiangtan, China; 4 UTA, Arlington, Texas, United States; 5 University of Southern Maine, Portland, Maine, United States

## Abstract

Objective: Using the stress-coping theory, the aims of the present study were to test what levels of caregiving intensity (hours actually spent on caregiving every day) posed the most negative influence on caregiver burden as well as how social support moderated such associations among dementia caregivers. Methods: Data from the baseline assessment of the Resources for Enhancing Alzheimer’s Caregiver Health (REACH II) (N = 637) were used. Caregiver burden (12-item Zarit caregiver burden scale), caregiving intensity (caregiving hours), and social support (Lubben social network, received support, satisfaction with support, and negative interactions) were the main measurements. Separate multivariate regression models were conducted with Stata 16. Results: The results showed that the relationships between caregiving hours and caregiver burden were nonlinear after controlling all of the socio-demographic variables. Further analyses showed that when caregiving hours reached 13.50 hours per day, the levels of burden were the highest. In addition, received social support, satisfaction with social support, and social network significantly moderated the relationship between caregiving hours and caregiver burden among dementia caregivers when they were examined separately. However, only social network played a significant moderator role when examining the four social support indicators simultaneously. Discussion and conclusion: These findings suggest the need for programs and practices on educating caregivers regarding how to identify, approach, and gain social support/s, especially in how to broaden the caregivers’ social network while caring for a family member with dementia.

